# Pharmacological Mechanism of Ganlu Powder in the Treatment of NASH Based on Network Pharmacology and Molecular Docking

**DOI:** 10.1155/2022/7251450

**Published:** 2022-06-29

**Authors:** Rui Gao, Xiaobo Zhang, Zhen Zhou, Jiayi Sun, Xuehua Tang, Jialiang Li, Xin Zhou, Tao Shen

**Affiliations:** ^1^School of Basic Medicine, Chengdu University of Traditional Chinese Medicine, Chengdu, China; ^2^Menzies Institute for Medical Research, University of Tasmania, Hobart, Tasmania, Australia; ^3^Innovative Institute of Chinese Medicine and Pharmacy, Chengdu University of Traditional Chinese Medicine, Chengdu, China; ^4^Academic Department, Chengdu Hemoyunyin Medical Laboratory Co., Ltd, China

## Abstract

Nonalcoholic steatohepatitis (NASH), a progression of nonalcoholic fatty liver disease (NAFLD), is a clinical syndrome characterized by liver steatosis, inflammation, and hepatocellular damage. Ganlu powder (GLP) is a classic traditional Chinese medicine prescription that has shown favorable treatment effects on NASH. However, the underlying therapeutic mechanisms are still poorly understood. This study is aimed at exploring the potential mechanism of GLP in the treatment of NASH via network pharmacology and molecular docking. PubMed and CNKI databases were used to identify the components of GLP. Swiss and STITCH databases were employed to obtain corresponding drug targets. NASH targets were adopted from the Therapeutic Target Database (TTD), DisGeNET, DrugBank, GeneCards, and MalaCards databases. Cytoscape software was utilized to construct “drug-ingredient-target-disease” networks and the protein-protein interaction (PPI) network of GLP in NASH. AKT1 was identified as the key target. The GO functional enrichment analysis revealed that GLP might treat NASH by modulating the inflammatory response and regulating phosphatidylinositol 3-kinase signaling. The KEGG analysis showed that GLP might treat NASH by regulating the tumor necrosis factor (TNF) signal pathway by affecting the role of AKT1. According to the network pharmacology results, a virtual docking of active compounds with AKT1 was carried out, and the results indicated that the 7 components, berberine, epiberberine, jatrorrhizine, coptisine, palmatine, evodiamine, and rutecarpine, can bind stably with AKT1 and have higher binding energy than AKT1 inhibitors. The overall study findings suggest that GLP may treat NASH by regulating AKT1.

## 1. Introduction

Nonalcoholic fatty liver disease (NAFLD) is one of the most common chronic liver diseases in the world [1]. It is a chronic metabolic disease caused by genetic and environmental factors and a hepatic presentation of metabolic syndrome. According to statistics, NAFLD is affecting approximately 20%-30% of the world population, which has become an increasingly serious public health problem [2, 3]. Nonalcoholic steatohepatitis (NASH), a severe form of NAFLD, is characterized by hepatic steatosis, inflammation, and hepatocyte injury. NASH can progress to cirrhosis and liver cancer and lead to poor clinical consequences [4, 5]. It was estimated that around 20% of NAFLD patients developed NASH in 10-15 years [6], and this rate is expected to rise to 27% in 2030, with NASH-associated all-cause mortality rising to up to 40% [7, 8]. Changes in lifestyle, such as regular exercise and developing healthy dietary habits, are primary strategies for managing NASH [9]. Notwithstanding this, it is often challenging for NASH patients to maintain a healthy lifestyle as it is often difficult for them to change their established living habits and the health benefits from lifestyle modifications often take a long time to emerge [10]. More importantly, lifestyle changes are frequently insufficient to improve patients' conditions, and pharmacological treatment is therefore needed.

Currently, there are no drugs approved for the treatment of NASH by the U.S. Food and Drug Administration (FDA). NASH is not unifactorial but a common multifactorial condition; thus, the effect of treatment for a single target is suboptimal [11]. Traditional Chinese medicine (TCM), which has been used in China for more than 2000 years, possesses the advantages of multicomponent, multitarget, and multipathway and thus has a great potential for improving hepatic steatosis and reducing inflammation [12, 13]. Ganlu powder (GLP), also known as Ganlu san or Coptis-Evodia herb couple, was a classic TCM formula initially recorded in *Sheng ji zong lu* and composed of two herbs, namely *Coptidis Rhizoma* and *Evodiae Fructus*, which have been mainly used for the treatment of gastrointestinal diseases including diarrhea, dysentery, and other inflammatory diseases. Pharmacological studies showed that GLP can lower blood lipids, improve inflammation, and regulate cholesterol metabolism. Zhang et al. [14] found that GLP is also effective for treating NASH. It can effectively improve the symptoms of NAFLD patients, reduce liver inflammation, improve liver steatosis, regulate glucose and lipid metabolism, and lower body weight and waist circumference of obese individuals, without raising apparent adverse reactions.

As with many other TCMs, the underlying mechanisms of GLP in treating NASH remain poorly understood. The nature of TCM makes it difficult for traditional methods to fully understand how these herbs with multiple active ingredients exert their synergistic biological effects through multiple targets and pathways in human beings [15, 16]. Network pharmacology is an emerging discipline proposed by professor Hopkins in 2007 [17]. This approach breaks the previous concept of a single ingredient/single target/disease and can thus be used as a useful tool in the studies of the “multicomponent, multitarget, and multibiological regulation function synergy” model of Chinese herbs [18, 19]. In this study, we studied the potential targets of GLP for NASH using network pharmacology and used the maximum degree value to identify the core targets in the gene network that indicate the role of the nodes in network pharmacology. DAVID database was used for enrichment analysis to determine the Gene Ontology and KEGG pathways that involved GLP. The results of network pharmacology were validated by virtual molecular docking, a computational chemistry technique based on the known ligand and receptor structure, used to simulate the binding interactions of bioactive components with core targets [20]. These analyses were able to provide valuable insight in understanding the molecular mechanisms of GLP in the treatment of NASH.

## 2. Materials and Methods

### 2.1. Bioactive Compounds and Target Fishing

Two databases, PubMed (https://pubmed.ncbi.nlm.nih.gov/) and CNKI (https://www.cnki.net/), were utilized for bioactive compounds screening. The chemical structure of the components was drawn by ChemDraw 15.0. Swiss ADME was used to predict the drug-like properties and gastrointestinal absorption of the obtained ingredients of GLP, and the ingredients that did not meet the criteria were excluded. In order to comprehensively obtain the targets of bioactive components of GLP, the collected ingredients were imported into STITCH and Swiss Target Prediction for target prediction. During STITCH retrieval, the species was set to “homo sapiens”; during the Swiss Target Prediction retrieval, the SMILES number of the corresponding components was uploaded for target fishing. Finally, the targets downloaded from the two databases were merged and deduplicated, after which the targets of GLP were obtained.

### 2.2. NASH Targets Acquisition

Five databases including Therapeutic Target Database, DisGeNET, DrugBank, GeneCards, and MalaCards were used for NASH targets screening; “nonalcoholic steatohepatitis” was used as the keyword for retrieval [21]. The obtained data from different databases were combined for analysis.

### 2.3. Drug-Compounds-Target-Disease Network Construction

The obtained targets of 7 compounds of GLP and NASH-related targets were merged together and delineated with the Venn diagram plotted by an online tool (http://www.bioinformatics.com.cn/plot_basic_proportional_2_or_3_venn_diagram_028). The intersection of GLP and NASH-related targets was derived. The “drug-component-target-disease” interaction was established through Cytoscape3.7.1. In the network, nodes represent the compounds of GLP and the targets of NASH, edges represent the relationship between each node, and the number of edges is defined as “degree.”

### 2.4. Protein-Protein Interaction (PPI) Network Construction

The STITCH database is a platform for exploring known and predicted interactions between compounds and proteins [22]. Seventy common targets of the drug and the disease were introduced into the STRING database, the organism was set as “homo sapiens,” and the confidence level was set to “high confidence (confidence score > 0.7).” The PPI interaction network was downloaded, and the degree of the nodes was analyzed using the R language (version 3.6.3). The topological properties of the network were calculated with the Cytoscape Network Analyzer plugin in Cytoscape 3.7.2.

### 2.5. GO and KEGG Enrichment Analyses

Gene Ontology (GO) is a widely used bioinformatic method for annotating genes, typically including biological processes (BP), molecular function (CC), and molecular functions (MF). KEGG is an important technique for analyses of functions between different genes. To better interpret the functions of those overlapped targets, GO and KEGG enrichment analyses were conducted using the Database for Annotation, Visualization, and Integrated Discovery (DAVID), and the top 20 terms with *p* value < 0.01 were plotted by bar graphs or bubble map [23].

### 2.6. Molecular Docking

Based on the results of network pharmacology, the three-dimensional (3D) structure of the core target was downloaded from the Protein Data Bank (PDB) database (http://www.rcsb.org/). Meanwhile, we searched the inhibitors of core targets from DrugBank and PubMed. The protein structure was processed using PyMOL (version 1.7.2.1) to remove excess ligands, including removing water molecules and excess Mg^2+^, Cu^2+^, and SO4^2-^. ChemDraw was then used to optimize small ligand molecules and save the files in a mol2 format. The standardized proteins and ligands were imported into AutoDockTools (version 1.5.6) and converted into a PDBQT format. Using the AutoDock Vina software (version 1.1.2), we ran a program for molecular docking using docking parameters; the complex conformation with the minimum binding energy and highest binding affinity for each molecule was selected for further analysis [24]. Generally, the binding energy < 0 kcal/mol indicates that the ligand can bind to the receptor protein, while the binding energy ≤ −5.0 kcal/mol reveals that the ligand and the protein have a good binding ability [25]. Finally, the docking result was visualized by PyMOL and Discovery Studio.

## 3. Results

### 3.1. Bioactive Ingredients and Corresponding Targets

By searching PubMed and CNKI, we obtained the ingredients of the two herbs of GLP. After the screening of absorption, distribution, metabolism, and excretion (ADME) properties, we retained 7 main ingredients (5 in *Coptidis Rhizoma* and 2 in *Evodiae Fructus*) including berberine, palmatine, jatrorrhizine, coptisine, epiberberine, evodiamine, and rutecarpine. The ADME properties of each component are shown in Table 1. Although the literature reports that there are some other components in Coptis and Evodia, they were not considered in this analysis due to their relatively low content. Afterwards, we obtained 32 targets from STITCH, 637 targets from SwissTargetPrediction, and 312 targets were retained after merging and deduplication.

### 3.2. NASH Targets Screening

“Nonalcoholic steatohepatitis” was set as a search term and subjected to these five databases: TTD, DisGeNET, DrugBank, GeneCards, and MalaCards, from which 0, 434, 0, 652, and 48 targets were obtained, respectively. 879 NASH-related targets were identified after mergence and duplicate removal.

### 3.3. GLP-Compounds-Targets-NASH Network

**Figure 1:**
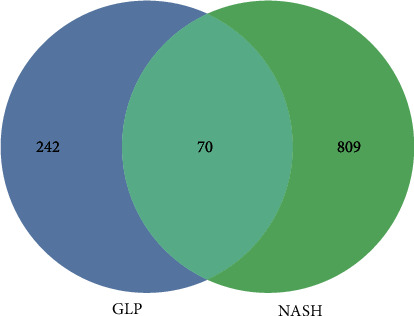
The 70 overlapped genes between NASH and GLP.

**Figure 2:**
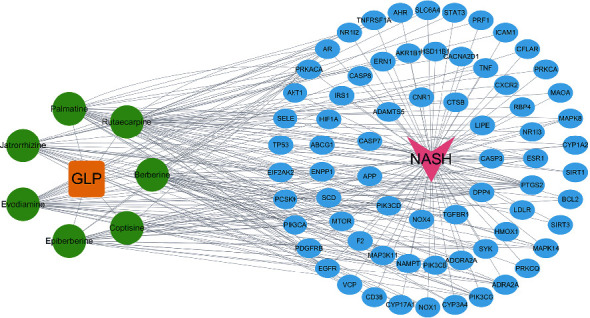
Drug-ingredient-target-disease network of GLP. The orange node represents drug, the green nodes represent ingredients, the blue nodes represent targets, and the red node represents disease (NASH).

After obtaining the GPL targets and the NASH targets, the Venn diagram was generated by mapping the intersection (Figure 1). The “GLP-compounds-targets-NASH” network was built with Cytoscape. In the complex network (Figure 2), the relationship between the identified active components and 35 targets is characterized by a total of 48 nodes and 44 edges.

### 3.4. PPI Network Analysis

**Figure 3:**
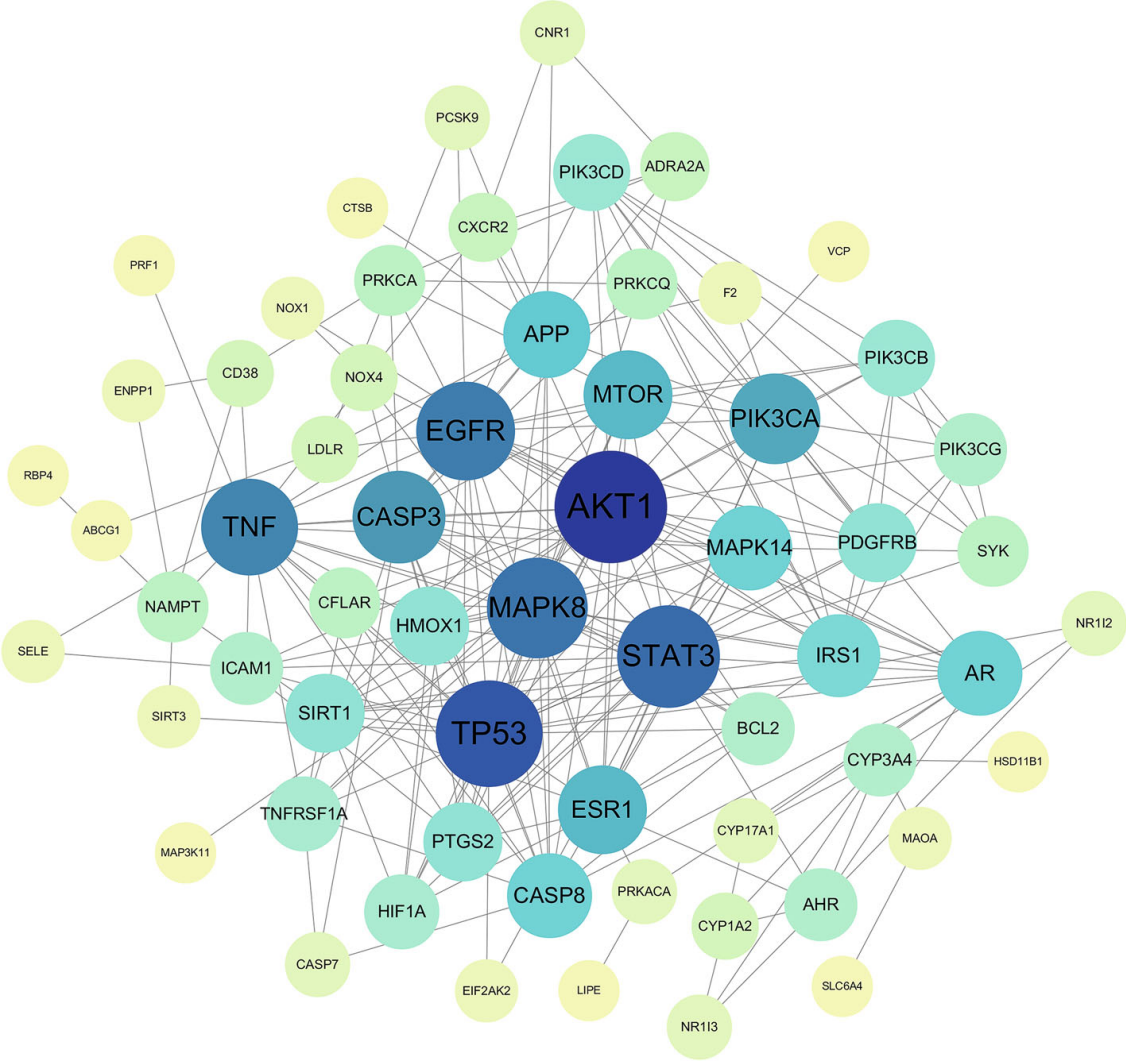
The protein-protein interaction (PPI) network.

**Figure 4:**
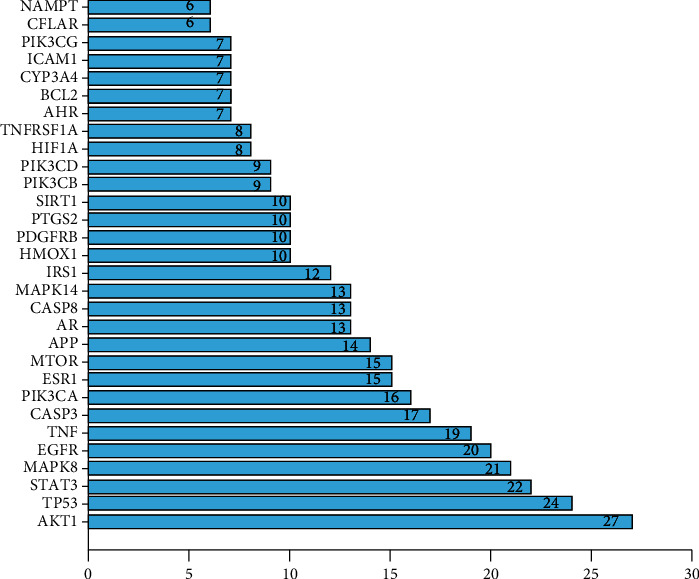
Ranking chart of the degree value of the PPI network. The *x*-axis represents the number of neighboring proteins of the target protein. The *y*-axis represents the target proteins.

Importing the 70 junction targets of GLP and NASH into the STRING database, we constructed a PPI network with 62 nodes and 235 edges (Figure 3). In the figure, the top 8 targets are AKT1, TP53, STAT3, MAPK8, EGFR, TNF, CASP3, and PIK3CA, among which AKT1 has the highest degree (Figure 4), indicating that it may be a key target of GLP in the treatment of NASH.

### 3.5. GO and KEGG Enrichment Analyses

**Figure 5:**
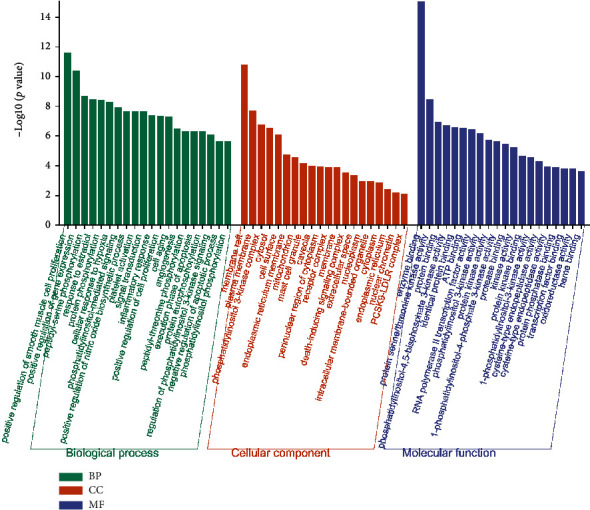
The Gene Ontology (GO) analysis of the 70 overlapping gene symbols associated with NASH. The *x*-axis represents the categories in the GO of the target genes, while the *y*-axis represents the *p* value (-log10) in the GO of the target genes.

**Figure 6:**
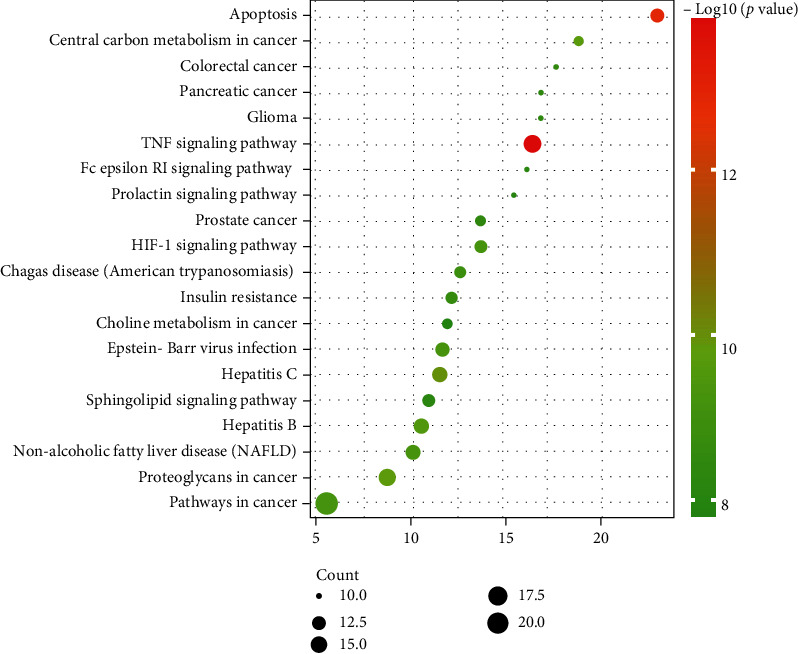
The KEGG pathway enrichment result of the 70 overlapping genes. The *x*-axis represents the fold enrichment of each pathway, the *y*-axis represents the main pathways (*p* value < 0.01), the size of the bubble indicates target counts in each pathway, and the color of the bubble indicates the *p* value.

**Figure 7:**
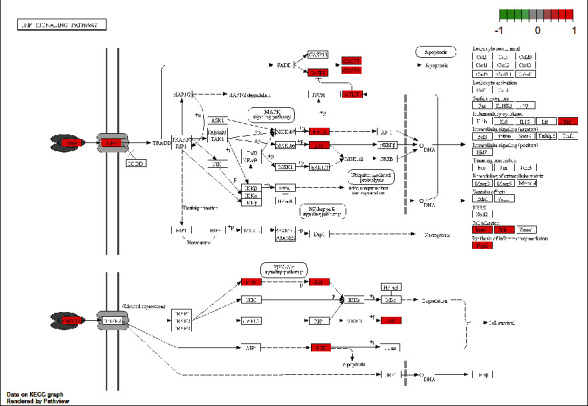
The KEGG pathway map of GLP treats NASH. The red nodes within the predicted signaling pathway represent the targets relevant with the corresponding pathway.

The DAVID web was used to perform GO and KEGG enrichment analyses of 70 intersected targets, the top 20 items with *p*-values <0.01 were visualized. In the BP category (Figure 5), the intersected genes were significantly enriched to protein phosphorylation, signal transduction, inflammatory response, protein autophosphorylation, regulation of phosphatidylinositol 3-kinase signaling, etc. The KEGG enrichment analysis revealed that the 70 intersected genes were highly related to NAFLD, HIF-1 signaling pathway, and insulin resistance, especially the TNF signaling pathway (Figures 6 and 7). Interestingly, AKT1 was involved in various processes.

### 3.6. Molecular Docking Evaluation

**Figure 8:**
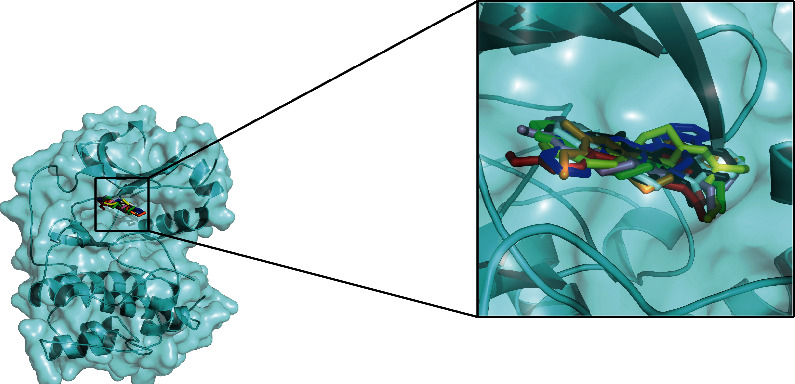
3D docking conformation of 7 ingredients and resveratrol with AKT1. In the figure, the red stick represents resveratrol, the green stick represents berberine, the yellow stick represents epiberberine, the pink stick represents jatrorrhizine, the blue stick represents coptisine, the orange-yellow stick represents palmatine, the white stick represents evodiamine, and the black stick represents rutecarpine.

**Figure 9:**
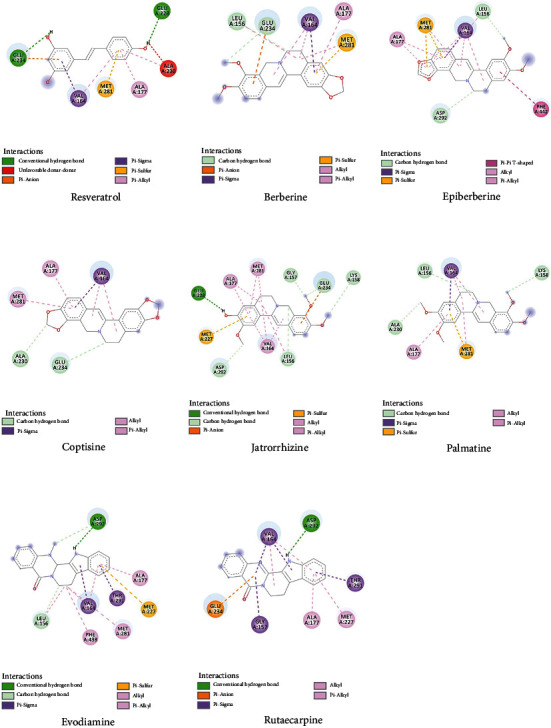
2D docking conformation of 7 ingredients and resveratrol with AKT1.

To further confirm whether the bioactive compounds of GLP can directly interact with the core target, the binding energy and dominant mode between the compounds with the key target were evaluated through molecular docking. The results show that all 7 components of GLP could be docked to AKT1 (PDB id: 3OCB), and they are located in the active pocket of the inhibitor resveratrol, which fits well in the docking position (Figure 8). The drug molecule and the protein receptor form various intermolecular forces, such as hydrogen bonds, C-H bonds, *π*-Alkyl, and other interaction forces (Figure 9). In addition, the 7 components have higher binding energy than the AKT1 inhibitor, resveratrol (Table 2).

## 4. Discussion

NASH is a metabolic stress liver injury strongly correlated with insulin resistance and genetic susceptibility, which can progress to liver cirrhosis and hepatocellular carcinoma. In addition, patients with NASH are at an increased risk of cardiovascular disease (CVD). The benefit of maintaining a healthy lifestyle is self-evident. The vast majority of patients, however, have difficulties in adhering to these healthy living habits, and the effect of behavioral changes on body weight and blood glucose management is often suboptimal. Due to the multiple and complex pathogenesis of NASH, there are no approved drugs for treatment. With the advancement of clinical medical research, the advantages of Chinese herbal medicine in the treatment of NASH have attracted substantial attention. As a historically old formula of TCM, the therapeutic effects of GLP on NASH attract our attention. In this study, 7 main active ingredients and 312 potential therapeutic targets of GLP, together with 879 NASH potential targets and 70 common targets, were screened using network analysis. As the complex network described, AKT1 is considered the core target of GLP in NASH treatment.

AKT1 is a member of the AKT kinase family, which regulates glycolipid metabolism, proliferation, and cell survival through a series of downstream substrates. Usually, the activation of AKT requires two key phosphorylation processes. Phosphoinositide-dependent protein kinase 1 (PDK1) initially phosphorylates AKT on threonine 308 (T308), an active phosphorylation site of AKT, leading to the activation process; mTORC2 then phosphorylates AKT on serine 473 to fully activate AKT [26].

In lipid metabolism, activated AKT induces the activation of mTOR, which subsequently recruits CRTC2 as a complex to promote SREBP-1 activity and adipogenesis. Yu et al. [27] found that transplanting the AKT plasmid into the mice through the tail intravenous injection can accelerate liver steatosis and inflammatory damage. AKT regulates fat synthesis factor SREPB-1 through the PI3K-AKT-mTOR signaling pathway, which increases the fatty acid synthesis and lipid content of liver cells and accelerates the NAFLD progression. In NASH animal models, it was also found that the PI3K-AKT signal was activated, leading to an aggravated liver damage. Inhibiting this pathway can markedly attenuate hepatocyte injury, inhibit hepatic stellate cell activation, and delay hepatic fibrosis progression [28, 29]. Nong and Chen [30] found that the Tiaogan Quzhi formula could improve the liver steatosis, inflammatory infiltration, and cell necrosis of NAFLD rats through the PI3K/AKT-mTOR signaling pathway. Similarly, Fan et al. found that another TCM formula, Tangganjian decoction, improves the hepatic glucose and lipid metabolism in rats with type 2 diabetes mellitus (T2DM) and NAFLD via activating the IRS/PI3K/AKT signaling pathway [31]. In general, AKT1 plays a crucial role in glucose and lipid metabolism and thus is a promising therapeutic target for metabolic diseases. The PPI result in our study revealed that GLP is effective in improving and treating NASH, possibly due to its regulation of AKT1.

According to the GO enrichment analysis result, the common targets of GLP and NASH were enriched to inflammatory response and regulation of phosphatidylinositol 3-kinase signaling, and AKT1 was also involved in these biological processes. Accumulating evidence has revealed that NASH is a metabolic inflammatory disease induced by lipid-oversupplied steatosis, and chronic inflammation is the main driver of NASH and one of the clinical features used to distinguish NASH from NAFLD [4, 32, 33]. In animal models of NASH, NF-*κ*B mRNA expressions in liver tissues were significantly increased. Other clinical studies also found that the NF-*κ*B activity was increased in NASH patients. AKT regulates NF-*κ*B signal transduction through phosphorylation of IKK*α*, which leads to the degradation of I*κ*B [34]. This process releases NF-*κ*B from the cytoplasm into the nucleus to further regulate the expression of downstream target genes and finally mediate multiple pathological processes such as liver inflammation and apoptosis. The evidence indicates that GLP may reduce liver damage by regulating the release of downstream inflammatory factors through AKT1.

KEGG enrichment results showed that the TNF signaling pathway was the most important signaling pathway, which involved the core target AKT1. TNF has two types: tumor necrosis factor-alpha (TNF-*α*) and tumor necrosis factor-beta (TNF-*β*). TNF-*α*, mainly secreted by activated macrophages, can promote the expression of proinflammatory factors and participate in systemic inflammation. Insulin resistance is involved in the development and progression of NASH. In NASH progression, TNF-*α* induces the activation of JNK, leading to the Ser site on the IRS protein in the phosphorylated insulin signaling pathway and consequently resulting in insulin resistance [35]. Our results showed that GLP could regulate the TNF signaling pathway through AKT1; thus, it might reduce insulin resistance in patients with NASH.

The protein-small molecular docking analysis showed that all 7 components of GLP were successfully docked to AKT1, located in the active pocket of resveratrol and fitting well in the active pocket. Meanwhile, the 7 components have better binding energy than the AKT1 inhibitor, resveratrol. The drug molecules and the protein receptors form multiple intermolecular forces, including hydrogen bonding, C-H bonding, *π*-alkyl hydrogen bonding, and other interactions. The docking score revealed that berberine, epiberberine, coptisine, evodiamine, and rutecarpine had higher interaction energy with AKT1 than resveratrol. This suggests that they were the most important active components contributing to the therapeutic effects of the GLP on NASH. It has been reported that berberine can induce autophagy by inhibiting mTOR, AKT, and MAPK (ERK, JNK, and p38) pathways [36]. Additionally, berberine induces autophagic death in acute lymphoblastic leukemia (ALL) cells by inactivating AKT/mTORC1 signaling [37]. Epiberberine is an alkaloid with low toxicity and exerts various activities including antiadipogenesis via AKT and ERK pathways [38]. In an in vitro experiment, epiberberine inhibits the phosphorylation of AKT, which then downregulates the major transcription factors of adipogenesis during adipocyte differentiation [39]. Coptisine can inhibit LPS-stimulated inflammation by blocking nuclear factor-kappa B, MAPK, and PI3K/AKT activation in macrophages, demonstrating its anti-inflammation properties. Evodiamine and rutecarpine, two alkaloids isolated from the unripe fruit of Evodia Fructus, were identified as two major active substances of Evodiae Fructus. Evodiamine inhibits cell proliferation by inducing cellular apoptosis via suppressing the PI3K/AKT pathway [40]. Moreover, evodiamine can significantly reduce the production of proinflammatory cytokines and inhibit the activation of inflammation-related pathways such as AKT, NF-*κ*B p65, ERK1/2, p38, and JNK [41]. Nie et al. [42] found that Rutecarpine ameliorates hyperlipidemia and hyperglycemia in fat-fed, streptozotocin-treated rats via regulating the IRS-1/PI3K/AKT signaling pathway. Although Nie et al.'s study did not report whether this bioactive compound directly inhibits AKT1, our docking result supports the possible protein-small molecule interaction.

## 5. Conclusion

In summary, this study tackles the molecular mechanism of GLP in the treatment of NASH based on bioinformatics and system pharmacology. Our findings indicate that multiple compounds in GLP may play a role in the treatment of NASH through the synergy of targets and signaling pathways. The active ingredients of GLP can act on the key gene target AKT1 and exert pharmacological effects through the TNF signaling pathway. Due to the limitations of biological calculation methods, future research with in vitro and in vivo experiments is needed to verify our findings.

## Figures and Tables

**Table 1:** The ADME properties of 7 components of GLP.

Compound	GI absorption	Drug likeness				
		Lipinski	Ghose	Veber	Egan	Muegge
Berberine	High	Yes	Yes	Yes	Yes	Yes
Epiberberine	High	Yes	Yes	Yes	Yes	Yes
Jatrorrhizine	High	Yes	Yes	Yes	Yes	Yes
Coptisine	High	Yes	Yes	Yes	Yes	Yes
Palmatine	High	Yes	Yes	Yes	Yes	Yes
Evodiamine	High	Yes	Yes	Yes	Yes	Yes
Rutecarpine	High	Yes	Yes	Yes	Yes	Yes

Abbreviations: ADME: absorption, distribution, metabolism, and excretion (ADME); GI: gastrointestinal; GLP: Ganlu powder.

**Table 2:** Molecular docking of 7 bioactive ingredients and the inhibitor with AKT1.

Compound	Structure	Receptor	Binding energy (kcal/mol)
Berberine	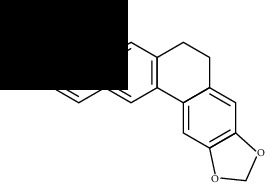	AKT1	-9.30
Epiberberine	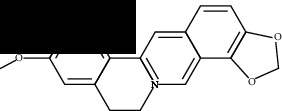	AKT1	-10.40
Jatrorrhizine	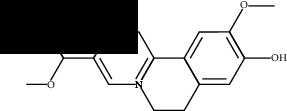	AKT1	-8.80
Coptisine	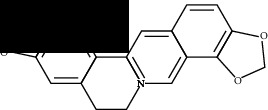	AKT1	-10.20
Palmatine	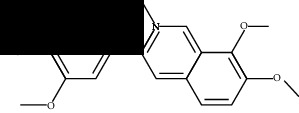	AKT1	-8.70
Evodiamine	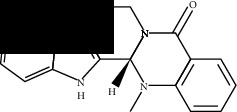	AKT1	-11.00
Rutecarpine	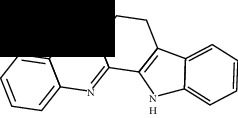	AKT1	-10.40
Resveratrol	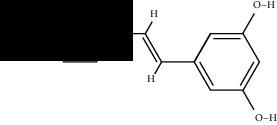	AKT1	-8.50

## Data Availability

The datasets used and/or analyzed during the current study are available from the corresponding author upon reasonable request.
